# The comparison of clinical effect, knee function, prognosis of double plate fixation and locking plate internal fixation for tibial plateau fractures

**DOI:** 10.12669/pjms.38.4.5340

**Published:** 2022

**Authors:** Qing Zhang, Jianzhong Zhao, Guangcheng Zhang, Jiazhu Tang, Wei Zhu, Mingjun Nie

**Affiliations:** 1 Qing Zhang, MD, Department of Orthopedics, Affiliated Hospital of Jiangsu University; No. 438, Jiefang Road, Zhenjiang, 212000, Jiangsu Province, P.R. China; 2 Jianzhong Zhao, MD, Department of Orthopedics, Affiliated Hospital of Jiangsu University; No. 438, Jiefang Road, Zhenjiang, 212000, Jiangsu Province, P.R. China; 3 Guangcheng Zhang, MD, Department of Orthopedics, Affiliated Hospital of Jiangsu University; No. 438, Jiefang Road, Zhenjiang, 212000, Jiangsu Province, P.R. China; 4 Jiazhu Tang, MD, Department of Orthopedics, Affiliated Hospital of Jiangsu University; No. 438, Jiefang Road, Zhenjiang, 212000, Jiangsu Province, P.R. China; 5 Wei Zhu, MD, Department of Orthopedics, Affiliated Hospital of Jiangsu University; No. 438, Jiefang Road, Zhenjiang, 212000, Jiangsu Province, P.R. China; 6 Mingjun Nie, MD, Department of Orthopedics, Affiliated Hospital of Jiangsu University; No. 438, Jiefang Road, Zhenjiang, 212000, Jiangsu Province, P.R. China

**Keywords:** Double plate fixation, Tibial plateau fracture, Locking plate internal fixation, Knee joint function

## Abstract

**Objectives:**

The purpose of this study was to investigate the clinical effect, knee function improvement and prognosis of double plate internal fixation and locking plate internal fixation in the treatment of tibial plateau fractures.

**Methods:**

Clinical data from 96 tibial plateau fracture patients treated at our hospital were analyzed retrospectively. Of these, 46 had been treated using locking plate internal fixation and 50 were treated with double T-shaped plate fixation. Clinically related indices, Hospital for Special Surgery (HSS) score of knee function, and ability of daily living (ADL) score were evaluated during postoperative follow-up.

**Results:**

No significant differences were observed in pre-operative patient characteristics in both groups. Healing time, time to weight-bearing, tibial plateau angle (TPA) and lateral posterior angle (PA) were all superior in the locking plate fixation group compared to the double plate fixation group. At three months post-operative visit, range of motion, knee function, flexion deformity, muscle strength, pain, and stability metrics were all superior in the locking plate fixation group compared to the double plate fixation group. ADL scores were also higher in the locking plate fixation group than in the double plate fixation group at three and six months follow-up.

**Conclusions:**

The clinical effect, knee function improvement and prognosis of locking plate internal fixation in the treatment of tibial plateau fractures are better than those of double plate fixation.

## INTRODUCTION

Tibial plateau fractures are mainly caused by high-energy injury^1^. The severe comminution of the articular surface often results in the involvement of the epiphysis or shaft of the tibial shaft, and may result in soft tissue injury, creating a more complex fracture situation.^2^ Tibial plateau fracture may lead to significant changes in the tibial morphology, such as collapse, splitting, compression, and similar phenomena.^3^ At present, the main method for the treatment of tibial plateau fractures is surgery. Double plate fixation and locking plate internal fixation are commonly used in clinic.^4^ However, which of the two methods is more efficacious is unclear. This study analyzed clinical data to examine the respective clinical efficacies of these two techniques, as well as their impacts on knee joint function and prognosis.

## METHODS

We retrospectively analyzed clinical data obtained from 96 tibial plateau fracture patients treated between May 2018 and March 2020. The Ethics Committee of the Affiliated Hospital of Jiangsu University approved this study (No: KY2021K0802, Date: 2021-08-16). Patient diagnoses was made using knee joint CT and MRI. Patients exhibiting good compliance were included while those with cognitive dysfunction, other fractures, and coagulation dysfunction were excluded. Among the 96 patients, 50 (26 males, 22 females), ranging in age between 26-56 years (average: 39.89±8.23 years), underwent double plate fixation. The other 46 patients (25 males, 23 females) with age ranging between 26-56 years (average: 39.60±7.94 years) underwent locking plate fixation.

Double plate fixation treatment had been performed as follows. After combined spinal epidural anesthesia, patient was guided to take the supine position. Double incisions were made on the posteromedial and anterolateral sides of the upper leg. The width of the two incisions was not less than 8cm. The fracture line of the medial and posterior metaphysis was fully exposed, and anatomical reduction was given until the force line of the medial column was restored. It was then fixed and a limited compression steel plate was selected. Anatomical reduction was performed until the force line of the medial column was restored. It was fixed using a limited compression plate. Afterwards, the lateral condyle of the tibia and knee joint was exposed by incision from the lateral side of the patella. The articular surface was reduced under direct vision and the lateral supporting T-shaped plate was fixed.

Locking plate internal fixation treatment was performed as follows: a double incision of 8-10 cm was made in the anteromedial and lateral sides of the knee. Then the joint capsule was cut to fully expose the joint surface, after which the joint surface was restored under direct vision or using a C-arm machine. Patients with bone defects needed iliac bone graft filling. After the normal axis of the tibia was restored, Kirschner wires were used for temporary fixation. After the reduction was satisfactory, a T-shaped or L-shaped locking plate was placed on the medial platform, while a golf locking plate was placed on the lateral platform to ensure good platform support. Hemostasis, washing, layer by layer suturing, and drainage tube placement was performed post-fixation in both groups, and antibiotics were given to prevent infection.

Postoperative follow-up and evaluation criteria included healing time, weight-bearing time, tibial plateau posterior angle (PA) and varus angle (TPA) at six months after operation. Three months post-operation, HSS score of knee function were retrieved from the medical records.^5^ HSS score evaluates range of motion, function, flexion deformity, muscle strength, pain, and stability, with a full score (100 points) indicating optimal recovery. ADL (activities of daily living) scores mainly include going up and down stairs, walking on the ground, going in and out of the toilet, eating, etc. ADL score ranges from 0 to100 points, with 0-20 points indicating complete dependence, 25-45 points indicating severe dependence, 50-70 points indicating moderate dependence, 75-95 points indicating mild dependence, and more than 95 points indicating independence.^6^

### Statistical Analysis:

All data were analyzed by SPSS 22.0, with measurement data expressed as x̄±SD. Comparisons between two groups were made using t-tests. F tests were used to examine two rates. P values below 0.05 were interpreted as statistically significant.

## RESULTS

No statistically significant differences were observed between the two patient groups in terms of gender or other demographic parameters (F = 0.041, P = 0.84), age (F = 0.031, P = 0.86), or BMI (F = 0, P = 0.986) (Table-I).

**Table-I:** Baseline patient characteristics.

Group	n	Gender(male/female)	Age(year)	BMI(kg/m²)
Double plate fixation group	46	26/22	39.89±8.23	23.08±1.14
Locking plate fixation group	50	25/23	39.60±7.94	23.08±1.13
F		0.041	0.031	0
P		0.840	0.860	0.986

Patients in the locking plate fixation group showed better clinical indicators such as healing time, shorter weight-bearing time, PA (°) and TPA (°), than patients in the double plate fixation group (P < 0.05) (Table-II).

**Table-II:** Comparison of clinical indicators.

Group	n	Healing time(w)	Loading time(w)	PA(°)	TPA(°)
Double plate fixation group	46	18.33±1.27	14.83±1.57	4.62±0.95	85.6±1.52
Locking plate fixation group	50	13.43±1.91	8.16±1.26	9.1±1.37	90.64±1.15
t		14.752	22.892	18.536	18.235
P		0.000	0.000	0.000	0.000

There was no significant difference in HSS score between the two groups before the operation (P > 0.05) (Table-III). However, three months after the operation, the HSS score in the locking plate internal fixation group were significantly higher than those in the double plate fixation group (P < 0.05) (Table-IV), There was no significant difference in ADL score between the two groups before the operation (P > 0.05); However, the ADL scores of locking plate fixation group were significantly higher than those of double plate fixation group at 3 and 6 months after the operation (P < 0.05) (Table-V).

**Table-III:** Comparison of preoperative HSS score of knee function.

Group	n	Activity	Function1	Flexion deformity	Muscle strength	Pain	Stability
Double plate fixation group	46	8.87±0.76	11.29±1.16	3.33±0.47	3.87±0.78	17.81±0.73	3.83±0.66
Locking plate fixation group	50	8.83±0.69	11.22±1.21	3.33±0.47	3.89±0.83	17.85±0.71	3.89±0.59
t		0.28	0.258	0	0.126	0.282	0.487
P		0.78	0.797	1	0.9	0.779	0.627

**Table-IV:** Comparison of HSS score of knee function at 3 months post-operation.

Group	n	Activity	Function1	Flexion deformity	Muscle strength	Pain	Stability
Double plate fixation group	46	14.06±1.74	16.08±0.76	5.95±0.82	5.62±0.7	20.75±0.66	5.79±0.61
Locking plate fixation group	50	15.27±0.76	18.83±0.81	8.18±0.76	7.7±0.65	24.75±0.83	7.95±0.65
t		4.398	17.102	13.758	15.061	25.854	16.731
P		0.000	0.000	0.000	0.000	0.000	0.000

**Table-V:** Comparison of pre-operative and post-operative ADL scores between the two patient groups.

Group	n	Before treatment	3 months after treatment	6 months after treatment
Double plate fixation group	46	58±4.66	69.14±3.08	80.58±2.17
Locking plate fixation group	50	58.5±4.57	78.64±1.78	88.66±2.39
t		0.531	18.499	17.339
P		0.597	0.000	0.000

## DISCUSSION

Incidence of tibial plateau fracture are on the rise in China due to an aging population and greater accessibility to personal motorized vehicles.^7^ The special position and physiological structure of the knee joint makes clinical treatment more difficult and knee joint function recovery slower, which seriously affects the quality of life of patients.^8^,^9^ Internal fixation is one of the most effective methods for the treatment of tibial plateau fractures. Most traditional operations use the median knee incision, which will fully expose the fracture section and block the blood flow in the anterior tibial area. In addition, fracture trauma and surgical operation will separate the soft tissue and makes it easy to skin flap infection and tissue necrosis.^10^

This study showed that locking plate and double plate internal fixations have achieved good results in the treatment of tibial plateau fractures. We show that the locking plate internal fixation results in better clinical efficiency compared to the double plate internal fixation. The use of locking plate internal fixation results in better stability. Compression fixation of the broken end of the fracture also has better clinical stability. It is more conducive to the early functional exercise of patients with complex tibial plateau fractures and promotes the recovery of knee joint ability.^11^ Yunfeng Yao et al.^12^ conducted a randomized controlled study on the surgical treatment of 86 patients with double condylar fractures of the tibial plateau. The results showed that both double plate and locking plate fixation had good effects, but locking plate not only provided stability similar to double plate, but also reduced operation time and soft tissue complications, contributed to fracture healing and shortened hospital stay.^13^

Since tibial repair operations entail a median incision of the knee, they result in the extensive stripping of soft tissue. This can easily result in complications such as delayed fracture healing and infection.^14^ Therefore, for tibial plateau fractures, internal fixation methods such as double plate fixation and locking plate fixation are often adopted.^15^ While double plate internal fixation can provide local stability and continuous fixation force to the knee joint in order to prevent fracture displacement and force line change, the technique is associated with a long healing time, which negatively impacts prognosis by delaying the return to normal activity.^16^,^17^ Medical technology developments have led to the gradual implementation of locking plate internal fixation in clinical practice. Locking plate internal fixation uses a self-tapping screw, eliminating the need for a bone drill or tapping during the fixation process. This can reduce the pressure between the bone cortex and the steel plate, thus promoting blood supply penetration and periosteal growth, leading to quicker recovery.^18^,^19^ Moreover, the internal fixation screw benefits from strong shear forces and does not detach easily, which further promotes fracture healing.^20^,^21^ This increased stability relative to double plate fixation is conducive to early initiation of functional recovery exercises, thus accelerating joint function restoration and improving prognosis.^22^ The results of this study also showed that the fracture healing time of locking plate internal fixation group was significantly shorter than that of double plate internal fixation group, and the recovery of knee function was also better than that of double plate internal fixation group.

### Limitations of the study:

It includes environment, and small sample size which might have an impact on the results. Moreover, there is no detailed analysis of the treatment effects, so further investigation is needed.

## CONCLUSION

Locking plate internal fixation is more effective for improving the clinical symptoms of tibial plateau fracture patients compared to double plate fixation. Patients undergoing locking plate internal fixation showed shorter fracture healing times, leading to earlier recovery of knee joint function and improved daily activity. This study can provide clinicians with some guidelines for selecting the optimal method for the treatment of tibial plateau fractures.

### Authors’ contributions:

**QZ:** Conceived and designed the study.

**JZ, GZ, JT and WZ:** Collected the data and performed the analysis.

**QZ:** Was involved in the Writing of the manuscript and is responsible for integrity of the study.

**MN:** Edited the manuscript.

All authors have read and approved the final manuscript.
